# Will All Scientists Working on Snails and the Diseases They Transmit Please Stand Up?

**DOI:** 10.1371/journal.pntd.0001835

**Published:** 2012-12-27

**Authors:** Coen M. Adema, Christopher J. Bayne, Joanna M. Bridger, Matty Knight, Eric S. Loker, Timothy P. Yoshino, Si-Ming Zhang

**Affiliations:** 1 CETI, Department of Biology, University of New Mexico, Albuquerque, New Mexico, United States of America; 2 Department of Zoology, Oregon State University, Corvallis, Oregon, United States of America; 3 Brunel University, Uxbridge, United Kingdom; 4 Biomedical Research Institute, Rockville, Maryland, United States of America; 5 Department of Pathobiological Sciences, University of Wisconsin, School of Veterinary Medicine, Madison, Wisconsin, United States of America

If this request had been made during the presidential address at the ASTMH meeting in Philadelphia in 2011, even though the room was filled beyond capacity, only a few people would have stood up. Yet, 300 million disadvantaged people suffer from snail-transmitted infections, with consequences ranging from life-threatening cholangiocarcinoma to subtle morbidity effects that stunt physical and mental development. The disability-adjusted life year (DALY) scores for these diseases have long been underestimated. The term “*neglected* tropical diseases” truly applies to all snail-borne infections, including schistosomiasis, fascioliasis, fasciolopsiasis, paragonimiasis, opisthorchiasis, clonorchiasis, and angiostrongyliasis [1]–[7]. The prevalence of most of the parasites involved has scarcely diminished in recent decades. The resilience of the snails that transmit them, such as *Biomphalaria* hosting *Schistosoma mansoni* in Africa, Yemen, or South America, or lymnaeid snails supporting *Fasciola hepatica* in Bolivia and elsewhere, provides a remarkable stability to the life cycles involved. Snail-borne infections provide a worthy challenge for any young parasitologist looking for an exciting career.

The recent World Health Organization (WHO) announcement of a global effort to eliminate human schistosomiasis by 2025 [8] is an inspiring clarion call that underscores the need for more emphasis on snail-related research. Future control of snail-borne parasites needs to be considered outside of the box of current, almost exclusive, reliance on chemotherapy. Although it is essential and surely must continue, chemotherapy alone may never achieve transmission control or elimination [9], and resistance is an ever-present possibility [10]–[12], especially when drug options are few, the extent of treatment is broadened, and the size of drug-sensitive parasite refugia diminishes [13].

So, how can study of relevant snails contribute to eliminating schistosomiasis and other snail-borne parasites? A detailed grasp of the role of snails in transmission is essential for developing *integrated* control strategies that also target the intramolluscan larval stages of parasites. For example, what determines the population structure and geographical distribution of snails that define endemic areas for parasite transmission, and how will global warming affect these [14]? To what extent is the number of infected snails dictated by immuno-compatibility between parasite and snail versus ecological factors that limit infections? Precise information *from the field* is lacking for how long infected snails continue to shed cercariae, and the number of cercariae produced per snail. Deciphering properties of immunity and virulence that have evolved to influence snail–parasite compatibility reveals determinants of host competence that will facilitate monitoring, predicting, and ultimately modifying transmission of schistosomiasis and other snail-borne parasites.

Exciting science can be done! Snails (and trematodes parasites) are lophotrochozoan protostomes, an animal lineage to which little attention has yet been paid—fundamental discoveries consequently lie ahead in a field that is not cluttered by many competing research groups. Recent novel basic insights into host–parasite interactions include the discovery of somatic diversification of immune molecules in invertebrates (*Biomphalaria*); the involvement of antigenic variation by *Schistosoma* to survive in snails; and the epigenetic modification of snail host chromosomes during the course of infection [15]–[17]. Much work is needed to clarify the mechanisms involved, work that can in today's difficult funding climate be justified by its applicability to alleviating the largely undiminished burden of snail-borne diseases. An excellent modern research toolkit justifies optimism that novel insights into *Biomphalaria*'s role in schistosomiasis transmission will be forthcoming. Microarray platforms [18], [19], next-generation sequencing [20], and RNA interference enable functional transcriptomic studies of *Biomphalaria* snails [21]–[23]. A draft assembly of the *B. glabrata* genome sequence is fully available [24], comprising the third component of the genome triad—human definitive host, parasite, and snail intermediate host—pertinent to schistosomiasis. The prospects for rapid development of similar tool kits for other important snail such as *Bulinus*, *Lymnaea*, *and Oncomelania* [25] are excellent.

Such new molecular capabilities have great potential for application to field investigations and disease control. These include identifying genetic markers for compatibility, developing sensitive means to detect transmission in areas subjected to control, and assessing receptors involved in chemoattraction of parasite to host. Next-generation sequencing can identify third party symbionts (bacteria or viruses) influencing snail-trematode interactions. The characterization of regulators of parasite transmission in natural snail population can contribute to the development of novel, ecologically friendly snail control methods (e.g., feeding or pheromone traps), and open up new lines of study such as introduction of snail transgenes capable of disrupting larval growth/differentiation. With so many snail-transmitted infections still at large, and so many obvious approaches awaiting investigation, we sincerely hope that the decline in snail-related funding, with a concomitant decline in the number of trained investigators, can be reversed. The availability of young workers even able to identify medically relevant snails has dropped to a shockingly low level.

To conclude, it is an unchanging reality that snails are essential for the continued flourishing of snail-borne parasites, including those that cause schistosomiasis. Given the recent call for global elimination of schistosomiasis, it is imperative we pursue a broader agenda that incorporates basic and applied snail research. From such efforts can emerge integrated and more sustainable control strategies. This will also help to arrest the alarming decline in young investigators, particularly in endemic countries. Given the considerable attention currently focused on other parasitic diseases such as malaria, could it be that the greatest opportunities to make significant new advances in parasitology now lie in other fields that have been truly neglected?
